# Theory-and evidence-based development and process evaluation of the *Move More for Life* program: a tailored-print intervention designed to promote physical activity among post-treatment breast cancer survivors

**DOI:** 10.1186/1479-5868-10-124

**Published:** 2013-11-05

**Authors:** Camille E Short, Erica L James, Ronald C Plotnikoff

**Affiliations:** 1 School of Medicine and Public Health, Priority Research Centre for Health Behaviour, University of Newcastle, Callaghan, Australia; 2 School of Education, Priority Research Centre for Physical Activity and Nutrition, University of Newcastle, Callaghan, Australia; 3 School of Human, Health and Social Sciences, Centre for Physical Activity Studies, Central Queensland University, Queensland, Australia

**Keywords:** Physical activity, Intervention development, Social cognitive theory, Breast cancer survivors, Tailored-print

## Abstract

**Objective:**

Several physical activity interventions have been effective in improving the health outcomes of breast cancer survivors. However, few interventions have provided detailed descriptions regarding *how* such interventions work. To develop evidence-based practice in this field, detailed descriptions of intervention development and delivery is needed. This paper aims to (1) describe the theory-and evidence-based development of the *Move More for Life* program, a physical activity program for breast cancer survivors; and (2) serve as an exemplar for theory-based applied research.

**Method:**

The program-planning model outlined by Kreuter and colleagues was used to develop the computer-tailored intervention.

**Results:**

The tailoring guide developed by Kreuter and colleagues served as a useful program planning tool in terms of integrating theory and evidence-based best practice into intervention strategies. Overall, participants rated the intervention positively, with the majority reporting that the tailored materials caught their attention, were personally relevant to them, and were useful for helping them to change their behaviour. However, there was considerable room for improvement.

**Conclusion:**

The *Move More for Life* program is an example of a theory-based, low-cost and potentially sustainable strategy to physical activity promotion and may stand as an exemplar for Social Cognitive Theory-based applied research. By providing a detailed description of the development of the *Move More for Life* program, a critical evaluation of the working mechanisms of the intervention is possible, and will guide researchers in the replication or adaption and re-application of the specified techniques. This has potential implications for researchers examining physical activity promotion among cancer survivors and for researchers exploring distance-based physical activity promotion techniques among other populations.

**Trial registrations:**

Australian New Zealand Clinical Trials Registry (ANZCTR) identifier: ACTRN12611001061921.

## Background

Breast cancer has the highest incident rate of any cancer among women in most regions of the world [1]. In developing countries, population-wide screening and the systematic use of adjuvant treatments has improved survival rates so that the majority of patients survive for at least 5 years following their diagnosis [1]. Whilst improved survival is duly welcomed, compared to the general population longer term survivors experience a survival deficit due to risk of recurrences, metastases and other chronic diseases [1]. Population health approaches aimed at reducing this survival deficit, as well as addressing other common physical and psycho-social issues experienced by survivors (e.g., reductions in quality of life, physical functioning and increased fatigue [2]) are needed.

One recommended approach is the promotion of regular physical activity (PA) [3]. The majority of breast cancer survivors are inactive or have difficulty maintaining activity levels over time [4,5]. Over 70 health outcome trials have documented the benefits of sustained PA among breast cancer survivors [6,7] and a growing body of observational research suggests that regular activity may also be a protective factor against poor survival outcomes, regardless of cancer stage [8-10]. Unfortunately, efforts to encourage regular PA are not yet a routine part of the cancer treatment or rehabilitation process [11-14] and population health approaches to promoting PA in this group are still needed [15,16].

Previous research in primary prevention suggests that computer-tailored print interventions, which utilise technology to provide individuals with health messages and behaviour change advice that is matched to their personal characteristics, may be a useful tool for improving public health. Computer-tailored print interventions have been shown to be more efficacious than other print-based approaches [17] and can personalise behaviour change advice at a relative low cost whilst maintaining wide reach, when compared to face-to-face interventions [16-18].

A major limitation of current research in the behaviour change field is the lack of scientific reporting regarding *how* interventions work. Current reporting typically focuses on ‘if’ and ‘how much’ the intervention works and has provided very little information regarding the theoretical basis of the intervention, the intervention techniques employed, and the links made between theoretical constructs and behaviour change techniques [19,20]. In the tailoring field, this has contributed to what is known as “the black box of tailoring” [21], whereby it is practically impossible to identify the working mechanisms of the intervention and build upon previous findings and methodologies. To address this issue, comprehensive guidelines have recently been published suggesting reporting standards for tailored interventions [21].

The current paper adheres to these reporting standards by describing the development and process evaluation of the *Move More for Life* intervention, a computer-tailored print intervention designed to promote PA among breast cancer survivors. The *Move More for Life* intervention was recently evaluated in a large Australian -based RCT with 330 post-treatment breast cancer survivors dispersed across the country. The current article aims to increase the public health impact of the *Move More for Life* intervention by offering insight into ‘how’ and for whom the intervention works, by providing sufficient detail to enable adaptation and/or replication of the intervention, and by providing recommendations for future research. Given the promise of computer tailored interventions for PA promotion [17] and the relatively high development time of these complex interventions, the information presented in this paper will accelerate the development process for other researchers developing similar interventions and reduce the likelihood of “reinventing the wheel”, which occurs all too often in the behaviour change field [20,21].

## Methods

Ethics approval for this research was obtained from the University of Newcastle Human Research Ethics committee (H-2010-11-3).

The intervention was developed using the 9-step program-planning model outlined by Kreuter et al. [22] (see Table 1). In brief, the 9 steps include: (1) analysing the health problem; (2) developing a program framework; (3) developing the tailoring assessment; (4) designing feedback; (5) writing tailored messages; (6) creating tailored algorithms; (7) automating the tailoring process; (8) implementing the program; and (9) evaluating the program. Actions undertaken in each step are described below.

**Table 1:** **The nine-step tailoring process (adapted from Kreuter et al. **[22]**)**

**Process**	**Aim of step**
1. Analysing the health problem	Understand the determinants of physical activity behaviour change
2. Developing a program framework	Outline the program “blue print” and develop a detailed description of each intervention component
3. Developing tailoring assessment	Develop assessment to measure participants status on key determinants
4. Designing feedback	Specify the design characteristics of the tailored material
5. Writing tailored messages	Outline unique characteristics of each message and write the tailored messages
6. Creating tailored algorithms	Link the specific responses to each assessment question with the corresponding message using logic statements – e.g. “if this, then that”
7. Automating the tailoring process	Assemble the computer program that generates tailored feedback
8. Implementing the program	Produce accurate and timely tailored feedback
9. Evaluating the program	Evaluate process, impact and outcome of the tailored program

### Step 1: Analysing the health problem

#### Reviewing applicable theories and models

The Theory of Planned Behaviour (TPB; [23]) and Social Cognitive Theory (SCT; [24,25]) were identified as the most popularly applied theoretical models to PA promption in cancer survivors. Both of these theories have shown to be useful frameworks for understanding the PA behaviour of cancer survivors [26], and there is evidence from meta-analyses that the use of these theories improves tailored-print health behaviour change intervention efficacy [27] and efficacy in psycho-oncology interventions [28].

#### Selecting theoretical framework

SCT was considered the most useful framework for informing the *Move More for Life* intervention for the following reasons: 1) a direct comparison of the two theories (in a non-diseased population) demonstrated that SCT accounted for greater variance in PA than TPB, mainly due to the predictive validity of self-efficacy [29]; 2) self-efficacy, a key construct in SCT, has shown to be an important correlate of PA in breast cancer survivors [30-32]; 3) interventions that utilise a greater number of SCT components have larger effect sizes than studies with fewer SCT components [28]; 4) previous research has shown that there is variability in breast cancer survivors scores on SCT constructs [33], which is a necessary criteria for tailoring; and, 5) unlike other models of health behaviour that are mainly concerned with predicting health habits, SCT offers both predictors and principles on how to inform, enable, guide, and motivate people to adopt habits that promote health [25]. The key constructs of SCT (adapted from [34]) are described in Table 2.

**Table 2:** Correspondence between social cognitive theory constructs and behaviour change techniques in the Move More for Life intervention

**Construct**	**Evidence-based intervention strategies**	**Move More for Life examples**
**Self-efficacy**		
Confidence in ability to engage in PA (task self-efficacy) and to overcome barriers to PA (barrier self-efficacy)	• Facilitate action planning [35]	• Activity at the end of each newsletter prompting participants to be specific about what, when and who they will be active with each week
	• Provide specific instructions [35]	
	• Reinforce efforts or progress towards goal behaviour [35]	
	• Provide feedback on participants past behaviour [36,37]	• Graphs in each newsletter displaying PA relative to the guidelines and past behaviour
	• Promote vicarious experience [37]	
		• Testimonial illustrating success
**Environment**		
External factors that influence (either positively or negatively) the PA behaviour of an individual	• Help secure social support in ways meaningful to individuals (note: planning social support and social change has been associated with lowering self-efficacy [35])	• Written advice encouraging participants to think of 1 or 2 people in their immediate circle they could share their physical activity plan with (to increase encouragement and opportunities for practical help).
	• Teach behaviour change skills that help individuals cope with environmental barriers e.g. time management [35]	• Provision of contact details for breast cancer specific PA groups
		• Encouragement to form a concrete plan
	• Provide individuals with PA resources and encourage links with the community [38]	
**Behavioural capability**		
Knowledge of what PA to perform and possession of PA skills necessary to perform those activities	• Inform breast cancer survivors of PA guidelines [39]	• Written feedback about whether or not participants are meeting the guidelines
	• Provide instructions on how to perform specific activities (e.g. stretching) [35]	• A3 poster illustrating stretches and resistance-based exercises
**Expectations**		
Expected effects of PA behaviour	• Address misconceptions about the benefits of PA and promote outcomes that have functional meaning for the individual (e.g. reducing fatigue, managing weight) [35].	• Provide overview of scientific evidence for the benefits of physical activity
		• Provide overview of how much other breast cancer survivors are exercising
		• Testimonial illustrating success
	• Facilitate social comparison [35]	
**Self-control**		
Personal regulation of goal-directed PA behaviour, includes activities such as goal setting, self-monitoring, problem solving and self-reward	• Promote self-regulation behaviours [40]	• A3 activity planner
	• Encourage participants to set PA challenges for themselves	
	• Encourage self-monitoring [36]	
**Observational learning**		
Learning from the experience of others, by watching the actions and outcomes of others PA behaviour	• Provide opportunities for vicarious experience via credible role models [34]	• Expert advice sections from exercise physiologist and behavioural scientist
		• Testimonial from breast cancer survivor

#### Reviewing previous literature

Reviews of computer-tailored interventions [27,41] provide strong support that tailored interventions are most effective when tailored using a mixture of: (1) social cognitive constructs; (2) demographic variables and (3) actual behaviour scores. In addition, audience segmentation research in the PA domain [42] suggests that broader health status factors beyond actual health behaviour (e.g., co-morbidities, level of disability) may also be important to consider when intervening in a tertiary prevention setting. That is, audience segmentation appears to be most worth-while when psycho-social and health status factors are combined together with demographic variables (which understandably have limited ability to distinguish between people in chronic disease groups but which ultimately enhance receptivity to and acceptance of health messages) [42].

Based on this information, we conducted a synthesis of the literature to: 1) identify which demographic and health behavioural variables should be targeted; and, 2) determine any social cognitive variables (either included or not included in SCT) that may be important to target in the intervention. Studies were identified through an electronic database search of all publication years (until Jan 2011) in Medline and Google scholar, using combinations of the following search strings: (Physical activit* or exercise) AND (correlate or determinant or mediator or moderator or intervention) AND (cancer survivor or breast cancer). Overall, we found there was limited research examining the determinants of PA behaviour change among cancer survivors. The majority of the studies identified were cross sectional (n = 20), with few longitudinal (n = 5) or intervention studies (n = 7) included. A summary of the findings are presented in Table 3. In brief, age, co-morbidities, weight status, and PA history were identified as potential factors that should be targeted for audience segmentation, whilst self-efficacy, social support, intention, and outcome expectations were highlighted as potential social-cognitive determinants of PA behaviour change. There were no social cognitive variables outside of SCT that were identified as essential to be addressed in the intervention. There was evidence that personality factors impact on PA participation [43-45], but this was not considered sufficient to warrant adding further complexity to the audience segmentation process. There was also strong evidence that PA intentions should be targeted. We chose to operationalize intentions as “proximal goals” to be consistent with the current conceptualisation of SCT [25].

**Table 3:** Summary table of literature exploring physical activity correlates and predictors among BCS

Demographics	
Income	• (+) *Cross-sectional.* Higher income associated with increased PA [33]
Age	• (+) *Longitudinal.* Younger age associated with lower PA post diagnosis [46]
	• (/) *Cross-sectional.* Age not associated with meeting the guidelines [47]
	• (/) *Intervention study.* Age not associated with exercise adherence [48]
Education	• (/) *Intervention study.* Education did not predict exercise adherence [48]
Marital status	• (/) *Intervention study.* Marital status did not predict exercise adherence [48]
Health status	
Co-morbidities	• (-) *Cross-sectional.* Higher co-morbidity associated with lower PA [47]
Weight	• (+) *Longitudinal.* Normal weight pre-diagnosis associated with less PA post-diagnosis [46]
	• (-) *Cross-sectional.* Higher BMI associated with reduced likelihood of exercising [47]
	• *Cross-sectional.* Lower sense of exercise self-efficacy among women who were overweight [49]
HRQL	• (+) *Cross-sectional .*Poorer HRQL was related to relapsing from active exercising to not exercising [50]
	• *Longitudinal.* HRQL (mental scale) significant predictor of rate of change of PA [51]
Fatigue	• (-) *Longitudinal.* Fatigue associated with lower PA at baseline but not associated with rate of change in PA [51].
Time since diagnosis	• (/) *Intervention study.* Time since diagnosis did not predict exercise adherence [48]
Stage of cancer	• (/) *Intervention study.* Stage of cancer did not predict exercise adherence [48]
Social cognitive	
Self-efficacy	• (+)*Cross-sectional.* Self-efficacy association with positive exercise changes [49]
	• *Cross-sectional.* Self-efficacy correlated with current PA levels independent of pre-treatment PA levels [31].
	• (+) *Cross-sectional.* Task self-efficacy highly predictive for both PA and exercise in the overall sample and in the subgroup of younger women. Barrier self-efficacy followed the same trend [47]
	• (+) *Intervention study.* Baseline self-efficacy significant predictor of mean minutes of weekly exercise and of meeting weekly goals. [48].
Social support	• (+) *Cross-sectional.* Having an exercise partner or role model associated with increased PA [33]
	• (+) *Longitudinal.* Family support predicts change in PA behaviour [51]
	• (+) *Cross-sectional.* Perceived social support related to increases in PA after diagnosis, even up to five years later [52]
	• (/) *Longitudinal.* Social support of friend (not exercise specific) not a predictor of PA at baseline [51]
Intention	• (+) *Cross-sectional.* Intention significantly predicted PA behaviour [53]
	• *Cross-sectional.* Intention explained 35% of the variance in exercise adherence [54]
Personality	• (+) *Cross-sectional.* Neurotic breast cancer survivors more like to relapse [43]
	• (+) *Intervention study.* Extraversion related to increased exercise [44]
	• (+) *Cross-sectional.* Optimism related to reports of increased exercise frequency in the past 6 months, although the amount of variance accounted for was small [45]
Perceived control	• (/) *Cross-sectional.* General locus of control unrelated to improvements in survivors PA [55]
Outcome expectation	• (+) *Cross-sectional.* Outcome Expectations significant predictor of PA and exercise in [47]
	• (+) *Mediation analysis.* Positive beliefs about PA and cancer recurrence are related to increased PA levels [56]
Decisional balance	• (/) *Cross-sectional.* Decisional balance did not predict exercise adherence [57]
Physical activity behaviour	
Pre-diagnosis PA level	• (-) *Longitudinal.* Women reporting more PA pre diagnosis had lower levels of PA post diagnosis [46]
	• (+) *Cross-sectional.* Prior exercise was a significant positive predictor of overall PA [47]
	• *Cross-sectional.* Direct association with Pre-treatment PA level and current PA level [31]
Baseline PA level	• (+) *Intervention study.* Baseline PA a significant predictor of mean minutes of weekly exercise [48]

#### Collecting original data

After reviewing the literature, we felt it necessary to gather further information about the application of SCT in the target group to inform our operationalization of SCT constructs and the appropriate selection of behaviour change strategies. We built upon Roger and colleagues [58] study with breast cancer patients and used a similar qualitative approach among post-treatment survivors. The findings of this research are described in detail elsewhere [59]. In brief, we found that motivation and health issues were the most common impediments for participating in regular PA. Among women who did participate, the immediate benefits of PA, such as weight loss and reducing fatigue were more salient motivators than preventing chronic disease. However, very few women were aware of the potential impact of PA on cancer recurrence. Participants’ demonstrated knowledge gaps in relation to what types of activity should be performed and how much activity is needed to achieve health benefits. Some women reported feeling pressured and judged when receiving PA advice. Preference for an exercise partner and the need for social support varied, with some women referring to themselves as “self-motivators” and preferring to exercise alone and others stating that having someone to answer to was a primary motivating factor for them. Although this work is preliminary and greater investigation on a population level is needed, the findings helped us to understand how each SCT construct may relate to PA behaviour in the post-treatment breast cancer population and ultimately set the ‘tone’ of the intervention (i.e., how the messages were framed).

### Step 2: Developing the program framework

#### Defining the program objectives

Program objectives were informed by the PA prescription guidelines for cancer survivors [3,60] and emerging evidence detailing the health risks associated with prolonged sedentary behaviour [5]. Specifically, the primary objectives of the program were to: (1) increase the total amount of minutes and days per week breast cancer survivors engage in health-enhancing PA (including aerobic and resistance-training) and: (2) promote maintenance of regular health-enhancing PA. The main secondary objective was to reduce the amount of time breast cancer survivors spend sitting in unbroken sedentary behaviour.

#### Defining program constraints

The project was constrained by a $52,000 (AUD) total project budget and a 3-year study timeline.

#### Designing the general program framework

##### Exploring factors impacting on intervention efficacy

The project team conducted a systematic review examining factors related to the efficacy of computer-tailored print interventions within the PA domain [17]. The findings of this review highlighted that the most effective interventions are those that contain multiple-contacts, are underpinned by theory or multiple theories (especially if the TTM [61] is used) and deliver print materials within 2 weeks of administering the tailoring assessment. Two relevant meta-analyses [27,41] were also identified. Both provided evidence that multiple-contact interventions are more likely to be effective than single-contact interventions, especially when messages are iteratively tailored (i.e., provide ongoing feedback based on updated participant data). One review [27] also provided evidence that tailored-print interventions delivered via newsletters have greater effect sizes than those delivered via other print mediums (e.g., booklets, letters). As such, we strived to develop a multiple-contact intervention that would offer iterative feedback and be delivered in a newsletter format.

##### Consulting with an expert advisory panel

One of the limitations of the tailoring literature is the lack of evidence regarding how many intervention contacts and at what frequency is optimal to facilitate behaviour change [17]. We formed an expert advisory panel to direct our decision making on these key aspects. The expert advisory panel consisted of a tailoring expert, an exercise physiologist specialising in breast cancer recovery, a consumer representative, and representatives from two breast cancer related support services. The key recommendations made by the advisory group were to ask participants directly what they find acceptable and to provide as many tailored materials as possible within the program constraints.

##### Exploring acceptability and program preferences among the target group

In line with the advisory groups recommendation, the acceptability of the proposed intervention was explored qualitatively among the eight post-treatment breast cancer survivors participating in the aforementioned interviews [59]. Participants were provided with a description of what computer-tailored written advice is, and were asked to provide feedback relating to the perceived usefulness of the intervention, interest in the intervention type and their preferences for number of contacts and intervention length. The key themes identified are summarised here together with illustrative quotes from participants.

##### Acceptability of the program

All of the participants supported the idea of a distance-based PA program designed to provide tailored-advice to individual breast cancer survivors. The main benefits identified by women were being provided with instructions on how to perform specific exercises correctly (e.g. stretches), having access to more information and having someone monitor their PA.

*I think that could be quite interesting. It’d be good to be told how to do those correctly, for instance like doing sit ups and um stretches that type of thing* (50 years old, 4 years post treatment).

*I think it sounds like a really good program. I reckon it’d help me to be more motivated and help with actually remembering to do stuff. Cause I usually go ohhh, I’ll do it tomorrow but if you’re actually monitoring it you’re more likely to actually do something* (44 years old, 5 years post treatment).

*I certainly know it’ll help me because besides my husband, I’m the only one really who is motivating me and it’ll be nice to see that somebody else is genuinely interested in how I’m progressing or what I’m doing* (55 years old, 3 years post treatment).

##### Preference for delivery schedule and program length

Preferences for the number and frequency of tailored newsletters was mixed. A few participants indicated receiving material once a month would be adequate, whereas others felt like it would be more useful to receive feedback weekly or fortnightly. Preference for delivery schedule was influenced by how active participants currently were, with participants who were less active requesting more support. A few participants acknowledged that the length of the assessment they would need to fill out to receive feedback would impact on their preference. There was a consensus amongst the participants that three months was an appropriate total program length.

*I think three months could be enough because after three months you should have got yourself into like a regular routine and you’re probably know what you’re doing by the three month mark, I would have thought. I think once a month is fine* (50 years old, 4 years post treatment).

*If it is just a quick five minute one than weekly would be fine but if it’s a sort of more ten, fifteen, twenty minute one probably more monthly* (44 years old, 5 years post treatment).

##### Deciding on the program framework

Drawing from the above information derived from the scientific literature, experts in the field, consumer representatives and the program constraints, we decided that the program framework would consist of three intervention contacts (computer tailored newsletters delivered via the mail) delivered over a 12 week period (6 weeks apart), and iteratively tailored based on ‘update cards’ (assessing PA and goal setting behaviour over the last month) sent to participants at 4 weeks and 8 weeks post-baseline. A detailed description of the program framework is published elsewhere [62].

#### Designing the feedback modules (i.e., newsletters)

This step involved mapping the theoretically-derived determinants (i.e., SCT constructs) to behaviour change techniques appropriate for use in a distance-based intervention [36,63]. Where possible, behaviour change techniques that have known efficacy (in terms of positive increases in PA and mediation effects) were chosen [35,36]. Findings from the qualitative research also informed this process. A description of the strategies used to address each SCT construct is provided in Table 2.

### Step 3: Developing a tailoring assessment questionnaire

The tailoring assessment questionnaire was imbedded within the baseline survey. All SCT constructs were assessed using validated and reliable measures where available. Some measures were adapted to make them more appropriate for use in the breast cancer population. A description of each measure is presented in detail elsewhere [62].

Given the recommendation to conduct mediation analyses when evaluating theory-based interventions [17,64] we strived to include measures that could be used for both tailoring and mediation analysis in the baseline and follow-up surveys. At times, this made the tailoring process more challenging (because the items were not purposely designed for tailoring) but was considered necessary to contain the length of the survey and therefore enhance completion. The demographic, social cognitive, health and behaviour variables measured in the baseline questionnaire were used to tailor messages in all three newsletters. Two update cards measuring PA and goal setting behaviour over the previous month were also utilised to provide iterative feedback in newsletters two and three.

### Step 4: Developing design templates

The design templates were developed in collaboration with a design firm (http://www.headjam.com.au/). We provided the design team with a draft of the newsletter layout based on a review of design features in previous interventions [17] and of current resources available to breast cancer survivors, such as the *Exercise for Health guidebook*[65] and the Breast Cancer Network Australia’s *Beacon* magazine (http://www.bcna.org.au/news/beacon-magazine). A simple layout was chosen for each newsletter to allow for easy navigation. Specifically, each newsletter consisted of four A4 (8.3 × 11.7 inches) pages and contained the following message blocks, respectively: a welcome message, targeted expert advice (non-tailored), feedback on PA behaviour and sitting time, a persuasive message (content-matched based on one or more SCT constructs) and an action planning task. The order in which SCT constructs were targeted was based on our literature review findings (e.g. self-efficacy and social support consistently related to PA behaviour) and Bandura’s [25] conceptual model regarding paths of influence (whereby self-efficacy is a focal determinant because of its effects on health behaviour both directly and indirectly via its influence on the other constructs e.g., self-control, outcome expectations and perceived facilitators).

**Figure 1:**
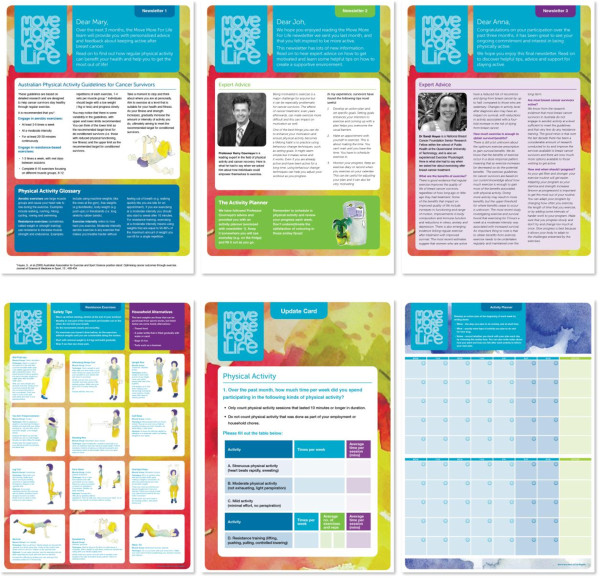
**Newsletters 1–3** ***, *** **exemplar exercise poster, update card and activity planner, respectively.**

The design team then developed the *Move More for Life* Logo and style (graphics style, text style, borders etc.), modified the draft templates for the three tailored newsletters, developed layout designs for the update card and additional resources (i.e., activity planner, exemplar exercise poster) and sourced all newsletter graphics. A personalised look was achieved by using water colour textures and hand-painted graphics (Figure 1). A description of each newsletter, along with the variables used to tailor information in each message block is published elsewhere [62].

### Step 5: Writing tailored messages

A message concept booklet was developed for each newsletter, whereby the intended message location, communication objective, message parameters (e.g., type of tailored message, message length, tailoring variables) and all possible feedback variables were outlined in detail before the writing process began. Messages were then written by CS, with a subset reviewed by EJ. The message concept booklets, including the tailored messages were then reviewed by a professional copy-editor to assure appropriateness and quality of the messages.

### Step 6: Creating tailored algorithms

This step involves linking the tailoring assessment items, responses and tailored messages using algorithms. Algorithms simplify the relation among these elements using three sets of variables, i.e., raw variables, intermediate variables, and feedback variables. They also help to identify all available response options from the tailoring assessment, establish priorities among these options, and indicate a default message in the event of a non-response [22].

#### Raw variable table

Raw variables represent participant responses from the assessment. The first step in creating the algorithms is generating a raw variable table, including the questions from the tailoring assessment and all possible responses (e.g., see Table 4).

**Table 4:** **Sample raw variable table from the *Move More for Life *** **study**

**Variable name**	**Description**	**Possible values**
R_first.name	Participants first name	20 characters
		Empty = not entered
R_PA1.AR.ST_Sess	Average number of sessions per week individual performed strenuous activity at baseline.	3 characters
		Empty = not entered
R_PA1.AR.ST_Min	Average time of each session (in minutes) individual spent performing strenuous activity at baseline.	5 characters
		Empty = not entered
R_PA1.AR.Mo_Sess	Average number of sessions per week individual performed moderate activity at baseline.	3 characters
		Empty = not entered
R_PA1.AR.Mo_Min	Average time of each session (in minutes) individual spent performing moderate activity at baseline.	5 characters
		Empty = not entered
R_diability1	Please rate how much your physical health limits your ability to engage in regular activity	1 not at all limited
		2 a little limited
		3 somewhat limited
		4 mostly limited
		5 completely limited
		Empty = not entered
R_PA.PrioD_AR_global	Has the amount of aerobic exercise you do changed since you were diagnosed with cancer?	1 No, I do the same amount of aerobic activity now
		2 Yes, I do more aerobic exercise more now
		3 Yes, I do less aerobic exercise now
		Empty = not entered

#### Intermediate variable table

The next phase involves creating an intermediate variable table, which allows the creator to form new variables from the raw data. For example, Table 5 demonstrates how we created an intermediate variable that describes whether or not participants are meeting the PA guidelines using the raw variables listed in Table 2. We created several other intermediate variables from the raw data, including intermediate variables for age, BMI, and for several of the SCT measures (e.g., high/low self-efficacy, high/low social support).

**Table 5:** **Sample intermediate variable from the intermediate variable table using in *Move More for Life***

**Variable name**	**Description**	**Formulas**
I_PA1.AR.Guid_modvig	Whether or not participants are meeting the aerobic guidelines of 30 minutes a day over 5 sessions at baseline (accounting for additional benefit of vigorous activity):	IF (R_PA1.AR.ST_Sess + R_PA1.AR.Mo_Sess < 5) AND (R_PA1.AR.ST_Min (x2) + R_PA1.AR.Mo_Min ≤ 150 THEN 1
	(1)Not meeting the guidelines	ELSE IF R_PA1.AR.ST_Sess + R_PA1.AR.Mo_Sess ≥ 5) AND (R_PA1.AR.ST_Min (x2) + R_PA1.AR.Mo_Min ≥ 150 THEN 2
	(2)Meeting the guidelines	

#### Feedback variable table

The final step involved the development of the feedback variables and related algorithms. In essence, the feedback variables, based on the raw and intermediate variables, define the specific conditions under which respondents receive particular messages. In the example within Table 6, the intermediate variable is combined with two raw variables (using logic statements) to indicate which PA message participants should receive based on particular responses to the tailoring assessment.

**Table 6:** Sample feedback variable from the feedback variable table used in Move More for Life

**Variable name**	**Algorithm**
F_Aerobic_performance1	IF I_PA1.AR.Guid_modvig = 1 AND R_diability1 = < 3 AND R_PA.PD_AR_global = 1 THEN 1
	ELSE IF I_PA1.AR.Guid_modvig = 1 AND R_diability1 = < 3 AND R_PA.PD_AR_global = 2 THEN 2
	ELSE IF I_PA1.AR.Guid_modvig = 1 AND R_diability1 = < 3 AND R_PA.PD_AR_global = 3 THEN 3
	ELSE IF I_PA1.AR.Guid_modvig = 1 AND R_diability1 = ≥3 AND R_PA.PD_AR_global = 1 THEN 4
	ELSE IF I_PA1.AR.Guid_modvig = 1 AND R_diability1 = ≥3 AND
	Etc.

### Step 7: Automating the tailoring process

The message concept booklets, design templates and variable tables were passed on to a computer programmer. The computer programmer built the tailoring program from scratch using Hyper Text Markup Language (HTML), Cascading Style Sheets (CSS) and the programming language JAVA. There were four stages to development.

#### Style

The design template was translated into a PDF by creating a print specific style sheet (CSS). A basic wireframe (i.e., a page schematic) was made and tested in three internet browsers after resetting all browser printing settings. The background and typography were then created and tested using copy from the design makeup. Firefox was selected as the best browser, as other tested browsers were unable to render multiple columns.

#### User interface

An online user interface, styled to match the baseline survey, was built using HTML to input data from the baseline survey and generate individual PDFs (i.e., tailored newsletters). Each field was assigned a tag.

#### Scripts

Scripts were built using JavaScript. Raw variables were matched to their corresponding tag and given a set of rules (if/else-if/else rules). When the variables match the rules the corresponding message block becomes ‘active’. This is achieved by changing the CSS display value from ‘none’ to the block value for the particular message.

#### Output

The final output, including all newsletters, was built into a HTML document. All scripts and messages options were included in line (i.e., not external).

##### Development costs

The tailoring system took the programmer three months to develop (1 month testing) and cost AUD $14,000. Overall, the *Move More for Life* program took eight (full-time) months to develop (inclusive of steps 1 & 6) and cost AUD $21,580. Development was carried out primarily by one researcher (CS), with regular support and guidance provided by EJ and RP. A detailed list of costs per stage of development, not including salary costs of project staff, is provided in Table 7.

**Table 7:** **Cost per development stage of the *Move More for Life *** **program**

**Component**	**Cost–time**	**Cost-$AUD**
Step 1		
Qualitative research	2 months	$1,800
Steps 2-7		
Newsletter design	2 month	$3,380
Copy-editing	1 month	$2,400
Computer programming	2 months	$14,000
Step 8		
330 Newsletters	1 month	$1381
250 Update cards	1 week	$221
380 Exercise example posters	1 week	$670
380 Activity planners	1 week	$670
3,000 Logo stickers	1 week	$500
Total	8 months	$21,022

Note: project team member’s time has not been costed.

### Step 8: Implementing the program

#### Promoting the program and recruiting participants

The tailored-print intervention was administered to 109 post-treatment breast cancer survivors recruited from around Australia via community and setting based recruitment methods (e.g., dissemination of study materials by cancer-focused organisations and health professionals; promotion of the study at breast-cancer specific community events). The overall recruitment index (mean number of days to accrue and enrol each participant) was 0.5.

#### Generating the tailored newsletters

For newsletter 1, participants were mailed the tailoring assessment and asked to return it within the next 10 days. On average, completed surveys were returned after 19 days (SD = 6.2). Participant data were entered into the online interface promptly, with newsletters generated (in pdf format) following receipt of the survey (M = 3.17 days, SD = 2.5). However, average time taken to print, package and mail the newsletters from receipt of participant data was 25 days (SD 4.9). This was due to the utilisation of a professional printing company that required all newsletters to be printed together, rather than individually once the pdfs were available. For newsletter two and three, if participant’s update cards were not returned within two weeks the newsletters were printed without iterative feedback. The majority of participants did return the update card within the specified timeframe for newsletter 1(70%) and for newsletter 2 (60%). However, only 49% of participants returned both update cards and 15% of participants did not return an update card at all.

### Step 9: Evaluating the program

#### Study sample and participant flow

Participants were mailed a questionnaire assessing their opinions of the intervention materials one month after receiving the final newsletter. Of the 109 participants who received the tailored intervention, 92 (84%) responded with feedback. There was no difference between responders and non-responders on important socio-demographic characteristics (i.e., age, marital status, aerobic activity, resistance activity, BMI, time-since treatment). Baseline characteristics of the 92 completers are presented in Table 8.

**Table 8:** Participant characteristics (n = 92)

*Demographics*	N	%
Age, years		
Mean	56	
Range	34-74	
Married, de facto	78	79.6
Completed University	47	46.8
Income > $1000 per week	37	37.7
Full-time employed	23	23.4
Born in Australia	74	75.5
Remote/regional location	50	51.0
*Health status*		
BMI, kg/m^2^		
Mean	26.60	
SD	5.11	
Months post (active) treatment		
Mean	40.91	
SD	39.01	
Disease stage		
0	3	3.06
1	21	21.43
2	29	29.59
3	22	22.45
4	2	2.04
unknown	21	21.43
Treatment		
Surgery	91	92.86
Chemotherapy	70	71.4
Radiotherapy	67	68.4
Hormones	55	56.1
*Physical activity status*		
Aerobic exercise > 150 min/wk + 5 or more sessions?	23	23.5
Resistance exercise > 6 exercises per week	15	15.31

#### Participant ratings of the tailored-intervention

The process evaluation assessment contained multiple choice questions (example item: ‘did the materials catch your attention?’) requiring participants to rate their response on a likert scale (e.g., ‘1 not at all’ – ‘5 very much’). The assessment also contained one open- ended question inviting participants to provide additional feedback. Overall, participants rated the intervention positively. The majority felt that the tailored materials caught their attention (74% responded ‘3 somewhat to 5 very much’; M = 3.6, SD = 0.88), were personally relevant to them (73% responded ‘3 somewhat to 5 very much’; M = 3.7, SD = 0.96), and were useful for helping them to change their behaviour (63.2% responded ‘3 somewhat to 5 very much’; M = 3.2, SD = 1.11). Given though that the intervention was designed to tailor information to individual characteristics it is of concern that one-quarter of the participants did not find the intervention personally relevant to them and one-third of participants did not find it useful for changing behaviour.

To gain a better understanding of why some participants did or did not rate the intervention highly we examined participant’s open-ended comments.

### Responses to open-ended questions

Several participants commented on the design of the newsletters. The majority of this feedback was positive, with participants describing the materials as eye catching, easy to read and colourful.

‘It was a great way to get info. Eye catching colours and easy to read info’.

‘Excellent materials: visual, colourful, well designed’.

‘I like very much the charts for the stretching exercises & resistance exercises. Shall keep them very handy’.

However, some participants found elements of the materials unwieldy in size and suggested alternative formats to improve useability.

‘I found the size unwieldy - didn't know where to put the charts. I would prefer an app’ .

‘Daily activity diary too big - better if it was purse-sized so you could carry it around and fill it in etc. Wallet sized cards with exercise plus health tips also would be good’.

Mixed feedback was provided about the content of the newsletters. Whilst some participants felt that the content was appropriate and has increased their awareness and helped them to understand their own limits, others felt that the information did not suitably acknowledge their personal limitations.

‘They made me realize how important exercise is to my wellbeing’ .

‘It was very useful in helping me realise what I could comfortably do plus what the type of activity was called’.

‘I felt some of the stretches plus exercises were not suitable for someone recovering from breast surgery with axillary clearance. The material introducing the exercises did not stress enough the importance of a gradual introduction to weights plus the drawings showed heavy weights which would be unnecessary or possibly damaging. More about a gradual build-up of weight plus repetition would be better’.

‘Personally I found the goal setting unrealistic. I could not fit any more physical activity into my week. I work almost full time, I have a 14 year old, a property, parents to look after, I have no more time left for more physical activity’.

Furthermore, to explore individual characteristics that may be related to how participants rated the intervention we conducted two ordinal logistical regression analyses; one examining the demographic, health characteristic and social-cognitive baseline variables associated with finding the intervention personally relevant, and a second examining the association between these predictors and finding the intervention useful. All variables were assessed using a self-report pen and paper survey. A detailed description of the measurement items and assessment protocol is provided elsewhere [62]. The results of the regression analyses are presented in Table 9.

**Table 9:** Individual factors associated with how participants rated the intervention materials

		**Personal relevance**		**Useful for changing PA**	
**Independent variable**	**Variable categories**	**OR ( 95% CI)**	**P value**	**OR (95% CI)**	**P value**
*Physical activity behaviour*					
MVPA (mins)		0.99 (0.99-1.00)	0.389	1.01 (1.00-1.01)	0.021*
Resistance training score		1.11 (1.03-1.19)	0.006^†^	1.12 (1.01-1.24)	0.024*
Sitting weekday (mins)		0. 99 (0.99-0.99)	0.035*	0.99 (0.98-0.99)	0.001^†^
Sitting weekend (mins)		0.99 (0.99-1.00)	0.439	0.99 (0.99-1.00)	0.998
*Demographics*					
Age		0.98 (0.84-1.12)	0.851	0.96 (0.81-1.14)	0.663
Marital status	Not married	7.08 (0.79-63.42)^×^	0.080	0.05 (0.00-0.94)	0.046*
Live with children	Yes	0.46 (0.72- 2.91)	0.410	0.026 (0.03-1.80)	0.175
Income	$1000+ per week	0.36 (0.06-2.06)	0.257	0.45 (0.06-3.45)	0.447
Employment	Not working	0.04 (0.01-0.33)	0.002^†^	1.39 (0.18-10.77) ^×^	0.748
Education	Secondary school	.	.	.	.
	Certificate or diploma	1.26 (0.11-13.88) ^×^	0.845	0.19 (0.01-2.98)	0.240
	University degree	0.65 (0.07-5.44)	0.694	0.07 (0.00-1.39)	0.083
Born in Australia	No	1.03 (0.16- 6.51)	0.973	0.43 (0.05-3.34)	0.428
PA Environment		0.95 (0.81-1.11)	0.556	1.10 (0.92-1.31)	0.269
Geographical location	Major City	6.08 (1.03- 35.56) ^×^	0.045*	0.97 (0.16-5.74)	0.974
*Psycho-social determinants*					
Outcome expectation		1.03 (0.86-1.21)	0.767	1.02 (0.84-1.23)	0.828
Outcome expectancy		1.07 (0.81-1.41)	0.622	1.42 (1.02-1.97)	0.034*
Task Self-efficacy		0.97 (0.79-1.18)	0.792	0.73 (0.56-0.96)	0.026*
Barrier Self-efficacy		0.99 (0.92- 1.07)	0.885	0.95 (0.88-1.03)	0.301
Family social support		1.02 (0.94-1.10)	0.600	0.92 (0.84-1.02)	0.129
Friend social support		1.01 (0.92-1.09)	0.858	1.03 (0.94-1.13)	0.446
Behavioural control		0.79 (0.65-0.97)	0.028*	0.79 (0.62-1.01)	0.058^~^
Self-regulation		1.07 (0.97-1.19)	0.153	1.04 (0.92-1.17)	0.475
Action planning		0.92 (0.72-1.17)	0.514	1.11 (0.84-1.47)	0.454
Observational learning		0.38 (0.15- 0.94)	0.038*	0.59 (0.17-2.05)	0.410
*Health/Cancer history*					
BMI		1.09 (0.89-1.34)	0.377	0.89 (0.70-1.14)	0.382
Quality of life (Fact-B)		0.99 (0.92-1.07)	0.968	1.06 (0.99-1.14)	0.069^~^
Fatigue (Facit)		1.07 (0.93- 1.22)	0.308	1.05 (0.93-1.18)	0.410
Time since treatment (months)		1.00 (0.98-1.03)	0.401	0.99 (0.96-1.01)	0.593
Radiotherapy	No	0.12 (0.02-0.72)	0.021*	0.47 (0.06-3.48)	0.463
Chemotherapy	No	1.93 (0.25-14.70) ^×^	0.523	0.84 (0.84-8.22) ^×^	0.878
Hormone therapy	No	18.89 (2.94-121.11 )^×^	0.002^†^	0.81 (0.14-4.64) ^×^	0.814

*P*^*~*^= marginally significant; P* = < 0.05; P^†^ ≤ 0.01; OR (CI)^×^*=* wide confidence interval (interpret with caution).

### Factors related to participants rating of the intervention

Individual characteristics associated with lower odds of rating the intervention as personally relevant were: higher self-reported sitting time, higher knowledge and skill for performing PA (behavioural control), higher opportunities for observing significant others engage in PA (observational learning), not working and having no history of radiotherapy. Factors associated with lower odds of finding the intervention useful were; higher levels of sitting time, higher knowledge and skills for performing PA, being unmarried and having higher confidence for performing PA(task self-efficacy; see Table 9).

Characteristics significantly associated with higher odds for rating the intervention personally relevant were having higher levels of resistance-training and living in a major city; factors associated with higher odds of rating the intervention useful were: higher levels of resistance-training, higher levels of aerobic activity, more positive outcome expectations and higher quality of life (see table).

Theories of information processing, such as the elaboration likelihood model [66], are often cited as the theoretical rationale for tailoring [22]. Such models suggest that personally relevant information is more elaborately processed then generic information and hence more likely to persuade an individual to change behaviour [66,67]. The above results suggest that there is some overlap between finding the intervention materials relevant and finding them useful. To explore this link further we ran an ordinal regression model and found that those who rated the intervention materials as personally relevant were 4.8 times more likely to rate the materials as useful for helping them to change their behaviour (OR: 4.8; CI: 3.3-7.12; *P* = 0.00). Randomised controlled trial findings exploring immediate and mid-term behavioural outcomes of the intervention as well as mediating mechanisms [62] are forthcoming [67] and will provide further insights into intervention efficacy and theoretical development.

## Discussion

Computer-tailored interventions are complex to develop. They necessitate extensive background research, deciding on a program framework, developing questionnaires and a corresponding message library containing hundreds if not thousands of messages, and computer technology to link all the components and automate the process. We found that the tailoring guide developed by Kreuter et al. [22] was a valuable resource for guiding this process and served as a useful program planning tool in terms of integrating theory and evidence-based best practice into intervention strategies. However, applying this framework was a time consuming process, mostly due to the lack of information available for selecting a behaviour change theory, the lack of synthesis regarding determinants of PA in the cancer literature and the limited and often non-descript reporting on how and why previously published interventions worked or did not work. Hence, this paper hopes to accelerate the development process of future interventions in this field, tailored or otherwise, by providing a detailed synthesis of the development process and by highlighting aspects of the intervention that could be improved. Lessons learned whilst implementing and evaluating the program are discussed below in combination with suggestions for future research.

### Lessons learned implementing the program

#### Producing timely tailored feedback

Our ability to produce feedback within a two week timeframe of receiving individual data was compromised by our printing protocol, which involved collating the newsletter pdf files (per newsletter) into a single file and sending it to a commercial printer. This added an extra 5–10 day delay to the delivery of all participant newsletters and an even longer delay for those participants who returned their survey and/or update cards well before the newsletters could be sent to print. Hence, the amount of time taken to deliver feedback to each individual was non-uniform and depended on how quickly individuals returned data to the research team. Newsletters could have been sent to participants in a more timely manner, within just a few days (M = 3.17 days, SD = 2.5), if printing had been conducted in-house. This approach is recommended for future print-based computer-tailored interventions.

Print-based delivery was chosen for the current intervention due to the perception that this would be most appropriate for the target group (i.e., older to middle aged women). However, we found that the majority of our participants did have internet access (97%) and several used email as their primary mode of contact with the intervention team. Hence, we suggest this assumption be revisited by future intervention developers targeting this group [17]. Researchers should consider other distance-based delivery modes, such as the internet and/or mobile devices. The major advantage of these technologically-based delivery modes is that they provide instant feedback and drastically reduce the data management tasks of the research team, which can be cumbersome in print-based interventions.

Population-based data examining intervention preferences, internet access and internet self-efficacy would be helpful to intervention developers when choosing a delivery mode. Similarly studies examining the relative performance of print and technologically-delivered computer-tailored interventions using the RE-AIM framework (Reach, Efficacy, Adoption, Implementation and Maintenance [16]) are recommended. Despite the public health potential of technologically delivered programs (e.g., web-based), preliminary findings suggest that current strategies for promoting adoption and retention in these interventions are limited in efficacy compared to print-based approaches [68].

#### Producing feedback that is personally relevant and useful for assisting behaviour

We were able to successfully provide personally relevant and useful information to the majority of the sample. However, it does appear that messages may not have been well-matched to some participants, particularly those with high scores on some psycho-social factors (i.e., confidence, knowledge and skill observational learning) and among those with potentially fewer resources (i.e., unmarried, not working, living outside of a major city). By conducting these analyses we have been able to pinpoint some potential weaknesses within the message library and suggest that future evaluations of tailored interventions include similar analyses to help refine tailored messages. It would also be useful to develop a tailoring system that has the capacity to provide information on the heterogeneity of the tailored messages actually delivered. This would allow researchers to examine if the tailoring algorithms used to select individual messages were appropriately designed to address the heterogeneity of the target group.

### Next steps for the Move More for Life Program

This article focuses on ‘how’ the *Move More for Life* intervention works and provides some preliminary process evaluation results describing ‘how much’ and for whom the intervention works. This work will be extended in future publications exploring: the efficacy of the intervention compared to another promising print intervention (described in detail elsewhere [65]) and a standard recommendation control group at short and medium term follow-ups; Social Cognitive Theory mediators of intervention effects at long-term follow-up; and moderators of intervention effects at both medium and long-term follow up. Further details regarding these analyses have been published elsewhere [62]. Combined, these articles ensure that a critical evaluation of the working mechanisms of the intervention is possible, and will hopefully guide researchers in the replication or adaption and re-application of the specified techniques.

### Consent

Written informed consent was obtained from participants for the publication of this report.

## Competing interests

The authors declare that they have no competing interests.

## Authors’ contributions

ELJ and CES conceived the study. All authors provided input into the study design. CES was primarily responsible for intervention design, with significant input from ELJ and RCP. CES, ELJ and RCP were responsible for drafting the manuscript. All authors critically evaluated the article for content and approved the final version.
